# Mesenchymal Stem Cell Secretome Attenuates PrP^106-126^-Induced Neurotoxicity by Suppressing Neuroinflammation and Apoptosis and Enhances Cell Migration

**DOI:** 10.3390/cells14110851

**Published:** 2025-06-05

**Authors:** Mohammed Zayed, Byung-Hoon Jeong

**Affiliations:** 1 Korea Zoonosis Research Institute, Jeonbuk National University, Iksan 54531, Republic of Korea; mzayed2@vet.svu.edu.eg; 2 Department of Bioactive Material Sciences, Institute for Molecular Biology and Genetics, Jeonbuk National University, Jeonju 54896, Republic of Korea; 3 Department of Surgery, College of Veterinary Medicine, South Valley University, Qena 83523, Egypt

**Keywords:** mesenchymal stem cells, prion disease, PrP^106−126^, apoptosis, conditioned medium, secretome, neurodegenerative diseases

## Abstract

Prion diseases are disorders caused by the misfolding of prion protein (PrP^Sc^), leading to the accumulation of an abnormal form of the normal prion protein (PrP) found in the host. The secretome of mesenchymal stem cells (MSCs), including paracrine-soluble factors, holds promising potential to stimulate host regenerative capability and alleviate organ disorders. In this research, our goal was to investigate the neuroprotective properties of the secretome derived from adipose-derived mesenchymal stem cells (AdMSC secretome) in relation to the toxicity caused by PrP^106−126^ in SH-SY5Y cells. The findings showed that PrP^106−126^ treatment exacerbated the neurotoxicity of SH-SY5Y cells, as indicated by increased lactate dehydrogenase (LDH) release. However, the AdMSC secretome significantly decreased LDH release. Under PrP^106−126^ stimulation, the AdMSC secretome downregulated inflammatory markers (*TNF-α* and *IL-1β*) and upregulated anti-inflammatory *IL-10*. Treatment with the AdMSC secretome markedly reduced GFAP immunoreactivity in astrocytic C8D1A cells compared to treatment with PrP^106−126^ alone. In addition, the AdMSC secretome reduced Iba-1 immunoreactivity in BV2 cells activated by LPS. Western blot analysis showed that the AdMSC secretome inhibited pro-apoptotic factor Bax induced by PrP^106−126^ and increased the expression of anti-apoptotic factor Bcl-2. However, no significant difference was observed in the expression of caspase-3. The AdMSC secretome exhibited a considerable migratory effect on SH-SY5Y cells after 24 h, as demonstrated by the scratch assay. The results suggest that the AdMSC secretome can attenuate PrP^106−126^-induced neuronal damage.

## 1. Introduction

Prion diseases are defined by the accumulation of abnormal prion protein (PrP^Sc^) in brain tissues and are marked by spongiform encephalopathy, neurotoxicity, and significant neuronal loss. The normal cellular prion protein (PrP^C^) converts into its pathogenic isoform, PrP^Sc^, which is noted for its β-sheet-rich structure, protease resistance, and tendency to aggregate [1,2]. These aggregates interfere with normal brain function, leading to the neurodegenerative symptoms observed in prion diseases [3,4,5]. Despite substantial investigation, the exact molecular pathways related to prion diseases are still not well understood. However, inflammatory cytokines, immune cells, and apoptosis are known hallmarks of prion disease pathogenesis [3,4,6]. The development of prion disease is greatly affected by neuroinflammation, which plays a crucial role in brain degeneration progression [7,8]. PrP^Sc^ induces the activation of various proinflammatory mediators in stimulated glial cells, which results in neuroinflammation [8]. As a result, immunohistochemical studies have shown that microglia are closely related to Creutzfeldt–Jakob Disease (CJD) [9]. In prion-infected animal models, the number of microglia is significantly higher than in non-prion controls [10]. In vitro studies have indicated that oxidative stress induced by microglia is a key factor in initiating neuronal cell death [11]. Furthermore, it has been shown that PrP^Sc^ accumulates within the astrocytes of both humans and rodents affected by prion diseases [12].

A neurotoxic peptide fragment that aligns with amino acids 106–126 of the human prion protein (PrP^106−126^) is frequently used as a relevant model for exploring the biological and physicochemical attributes of PrP^Sc^ [13]. Apoptosis can be triggered in vitro by exposing neuronal cells to PrP^Sc^ or PrP^106−126^ [11]. It has been shown that PrP^106−126^ induces apoptosis via mitochondrial disruption in the SH-SY5Y cell line [14]. Several studies using cell culture models have demonstrated that PrP^106−126^ induces neuropathological effects similar to those of PrP^Sc^ [15]. The activation of microglia and the subsequent release of pro-inflammatory cytokines [16] highlight the critical role of cell death in the progression of prion diseases.

At present, there is no effective treatment available for prion diseases. Mesenchymal stem cells (MSCs) are characterized by their multipotent, self-renewing capacity and active secretion of a broad spectrum of trophic factors under normal and hypoxic conditions [17,18,19,20,21]. Consequently, MSCs have been extensively researched as potential treatments in various prion disease models [22,23]. However, the specific mechanisms by which MSCs carry out their effects in prion diseases remain inadequately understood [22,23,24]. The initial assumption was that MSCs primarily functioned by differentiating into the necessary cell type and replacing damaged cells. However, recent studies have reported low survival rates and poor differentiation of transplanted MSCs [25,26]. The long-lasting beneficial effects of MSCs seem to be at odds with their short lifespan [27], indicating that the released paracrine factors may play a role in functional recovery [28]. MSCs are recognized for releasing a wide array of trophic and immunomodulatory factors, which can be collected in serum-free conditioned media, often referred to as the secretome [29]. In this secretome, MSCs release proteins, cytokines, and extracellular vesicles (EVs) that have been demonstrated to directly modulate inflammation, neurogenesis, and tissue repair [30]. Furthermore, MSCs release mitochondria, which play a crucial role in regulating the repair process [31,32]. These biomolecules can influence the tissue microenvironment and enhance intercellular communication, thereby supporting tissue repair and regeneration.

Studies have demonstrated that the secretome derived from cultured adipose-derived MSCs (AdMSC secretome) presents protection against oxidative stress in neurodegenerative diseases, including prion diseases [33,34,35]. The secretome of MSCs can also influence immune responses and reduce tissue damage caused by inflammation [36,37]. Recent research has shown that the conditioned media of MSCs significantly contributes to the suppression of microglial activation, helping to alleviate neuroinflammation in amyotrophic lateral sclerosis [38]. Thus, the MSC secretome could be a potential therapeutic approach for prion disorders. Nevertheless, although AdMSCs have shown significant promise in treating neurodegenerative diseases [22], it remains unclear how their secretome regulates inflammation and apoptosis induced by PrP^106−126^. Here, we focused on the effects of the AdMSC secretome against PrP^106−126^-induced inflammation and apoptosis in SH-SY5Y cells.

## 2. Materials and Methods

### 2.1. Cell Culture of AdMSCs and Preparation of the Secretome

All animal experiments were conducted in compliance with ethical guidelines approved by the Animal Care Committee of Jeonbuk National University (Protocol No. CBNU 2020-080) [35,39]. AdMSCs were isolated from the epididymal fat of 6–8-week-old male C57BL/6J mice (n = 5; Nara Biotech, Seoul, Republic of Korea) using established protocols with minor modifications [35,39]. Briefly, isolated AdMSCs were cultured in Dulbecco’s Modified Eagle Medium (DMEM; Gibco, Grand Island, NY, USA) supplemented with 10% fetal bovine serum (FBS; Thermo Fisher Scientific, Waltham, MA, USA) at 37 °C under 5% CO_2_. AdMSCs were characterized by their adherence to culture dishes, expression of MSC-specific surface markers, and multilineage differentiation potential, as validated in prior studies. The secretome-containing medium from AdMSCs was prepared as previously described [35]. Briefly, AdMSCs were first cultured in serum-containing medium for 48 h. The medium was then switched to serum-free medium, and after an additional 48 h, the conditioned medium was collected and concentrated using an Amicon Ultra-15 centrifugal filter unit (Merck Millipore, Burlington, MA, USA). The presence of bioactive molecules was confirmed using a mouse cytokine ELISA plate array (Signosis, Santa Clara, CA, USA).

### 2.2. Preparation of PrP^106−126^ Peptide and Cell Culture

The PrP^106−126^ peptide was synthesized, dissolved, and preserved as previously described [35,39]. Human neuroblastoma SH-SY5Y cells (ATCC CRL-2266; obtained from the Korean Cell Line Bank, Seoul, Republic of Korea) were maintained in DMEM/F12 medium (Gibco) with 10% FBS under standard culture conditions (37 °C, 5% CO_2_). Based on earlier studies, we fine-tuned the conditions using varying concentrations of PrP^106−126^ (0, 100, and 200 µM) and discovered that treatment with 100 µM PrP^106−126^ for 24 h led to significant neurotoxicity [35,40].

### 2.3. Release of Lactate Dehydrogenase (LDH)

SH-SY5Y cells (1 × 10^4^ cells) were plated and grown on a 96-well plate for a duration of 24 h. Cells were divided into four groups; control (no treatment), AdMSC secretome-treated (5 µg/mL), PrP^106−126^-treated (100 μM), and PrP^106−126^ co-treated with the AdMSC secretome at 37 °C and 5% CO_2_ for 24 h. To measure the release of LDH in the cultured medium, the manufacturer’s instructions were followed using the LDH Cytotoxicity Assay Kit (Antibodies.com, Cambridge, UK). In brief, 100 µL of the culture media was transferred to a fresh 96-well plate. After adding 100 μL of reaction solution to each well, the plate was incubated for 30 min at 37 °C. The measurement of LDH release was conducted by quantifying the absorbance at 565 nm using a microplate reader (Molecular Devices, San Jose, CA, USA). The data are presented as the mean ± SD from triplicate experiments.

### 2.4. Real-Time Polymerase Chain Reaction (RT-PCR)

To assess the anti-inflammatory effect of the secretome, PrP^106−126^ and the secretome were added to SH-SY5Y cells for 24 h. Untreated cells were used as a control. Total RNA was isolated using TRIzol reagent (Invitrogen, Waltham, MA, USA). The reverse-transcribed products were amplified using the SYBR method with the CFX96 real-time PCR system (Bio-Rad, Hercules, CA, USA) in accordance with the manufacturer’s guidelines. To examine the expression of mRNA, real-time PCR amplification of primers for pro- and anti-inflammatory markers (*TNF-α*, *IL-1β*, *IL-10*, and *IDO*) was performed (Table 1).

### 2.5. Immunofluorescence of Cells

Murine astrocyte C8D1A cells and murine microglial BV2 cells were cultured in DMEM/F12 medium (Gibco) supplemented with 10% FBS and 1% penicillin-streptomycin at 37 °C in a 5% CO_2_. Astrocyte C8D1A cells and BV2 cells were seeded in chamber slides (SPL Life Sciences Co., Pocheon, Republic of Korea) at a density of 2 × 10^4^/mL. The cells were subjected to treatment with PrP^106−126^ or LPS (1 μg/mL) with or without the AdMSC secretome and incubated for 24 h. The cells were fixed, permeabilized with 0.1% Triton X-100, and blocked with Power Block (BioGenex Laboratories, Fremont, CA, USA) for 30 min at room temperature. Primary antibodies against GFAP and Iba-1 were applied overnight at 4 °C, followed by incubation with secondary antibodies for 1 h at room temperature. Nuclei were counterstained with DAPI using ProLong Gold Antifade Mountant (Invitrogen). Fluorescence imaging was performed under standardized conditions using a fluorescence microscope (Zeiss Axio-Imager M2, Oberkochen, Germany), and fluorescence intensity was quantified using ImageJ software (v1.54p, NIH, Bethesda, MD, USA).

### 2.6. Western Blot Analysis

Following treatment, SH-SY5Y cells were lysed using RIPA lysis buffer (Thermo Fisher Scientific) along with a protease inhibitor cocktail (GenDEPOT, Baker, TX, USA). Protein concentrations were determined using the Coomassie Bradford protein assay kit, with bovine serum albumin (Thermo Fisher Scientific) serving as a standard. An equivalent quantity of protein (40 µg/well) was separated on a 12% SDS-polyacrylamide gel before being transferred onto a nitrocellulose membrane (Amersham, Little Chalfont, UK). The membrane was blocked with 5% skimmed milk before being incubated with primary antibodies against Bax and Bcl-2 (sc-7480 and sc-7382, respectively, Santa Cruz Biotechnology, Dallas, TX, USA), caspase-3 (#9662S, Cell Signaling Technology, Inc., Danvers, MA, USA), and β-actin (sc-47778, Santa Cruz Biotechnology) overnight at 4 °C. After washing, the membrane was incubated with a specific secondary antibody at room temperature for 1 h. An ECL western blot detection system was used to visualize the membranes following the manufacturer’s guidelines. Quantification of band intensities was performed using ImageJ gel analysis software. All procedures were replicated three times.

### 2.7. Scratch Assay

In the scratch assay, the migration of SH-SY5Y cells was evaluated using a monolayer wound assay, as previously described [39]. SH-SY5Y cells were plated in 24-well plates with DMEM supplemented with 10% FBS until they reached 80% confluence. A 200 µL pipette tip was used to scrape the cells across the plate. The cell culture medium was then immediately replaced with standard DMEM supplemented with different treatments. To evaluate cell migration, the cells were examined under a phase-contrast microscope at 0, 12, and 24 h. The area of wound closure was determined by calculating the ratio of the culture area after 24 h of migration to the culture area at the initial time point.

### 2.8. Statistical Analysis

Data are expressed as the mean ± standard deviation (SD). Statistical analyses were conducted using one-way analysis of variance (ANOVA) followed by Tukey’s post hoc test for multiple comparisons in SPSS 26.0 software (IBM, Armonk, NY, USA).

## 3. Results

### 3.1. The AdMSC Secretome Preserves the Viability of SH-SY5Y Cells

AdMSCs derived from adipose tissue in the inguinal region exhibited typical MSC morphology, surface marker expression, and trilineage differentiation potential [35,39]. Our previous analysis showed that the AdMSC secretome contains a higher concentration of different cytokines that play important roles in neuroprotection through their anti-inflammatory and anti-apoptotic activities, as previously reported [35,36,41].

**Figure 1:**
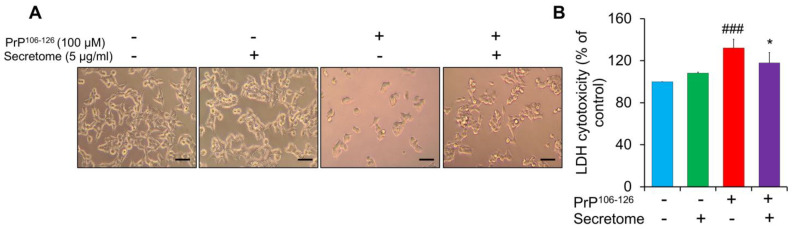
Effect of the adipose-derived mesenchymal stem cell (AdMSC) secretome on the viability of SH-SY5Y cells. (**A**) The morphology of SH-SY5Y cells was observed after exposure to 100 µM PrP^106−126^ or co-treatment with the AdMSC secretome (5 µg/mL). Scale bar = 100 µm. (**B**) LDH assay showing the effects of the AdMSC secretome on LDH release after 24 h of co-treatment with PrP^106−126^. Statistical analysis was performed using one-way ANOVA with Tukey’s post hoc test. Results are presented as mean ± standard deviation; n = 3 per condition. ### *p* < 0.001 vs. (control non-exposed group), * *p* < 0.05 vs. PrP^106−126^-treated group.

Consistent with earlier studies, PrP^106−126^ was widely used to induce neurotoxicity in various neuroblastoma cell lines [13,40]. Our previous findings showed that a 24 h exposure to 100 μM PrP^106−126^ notably reduced cell viability [40]. In the current study, SH-SY5Y cells were treated with PrP^106−126^ at 100 μM for 24 h to establish neurotoxicity. As shown in Figure 1A, the number of SH-SY5Y cells decreased following treatment with PrP^106−126^, whereas treatment with the AdMSC secretome alone did not adversely affect cell viability. The LDH assay, a typical indication of reduced cell integrity, was conducted to further assess the potential protective effects of the AdMSC secretome against PrP^106−126^-induced neurotoxicity. A significant increase in LDH release was noted in cells treated with PrP^106−126^ (*p* < 0.001) (Figure 1B). However, addition of the AdMSC secretome effectively reduced LDH release (*p* < 0.05) (Figure 1B). Overall, these findings indicated that the AdMSC secretome confers protection against PrP^106−126^-induced neurotoxicity in SH-SY5Y cells.

### 3.2. AdMSC Secretome Suppresses Reactive Astrocytes

**Figure 2:**
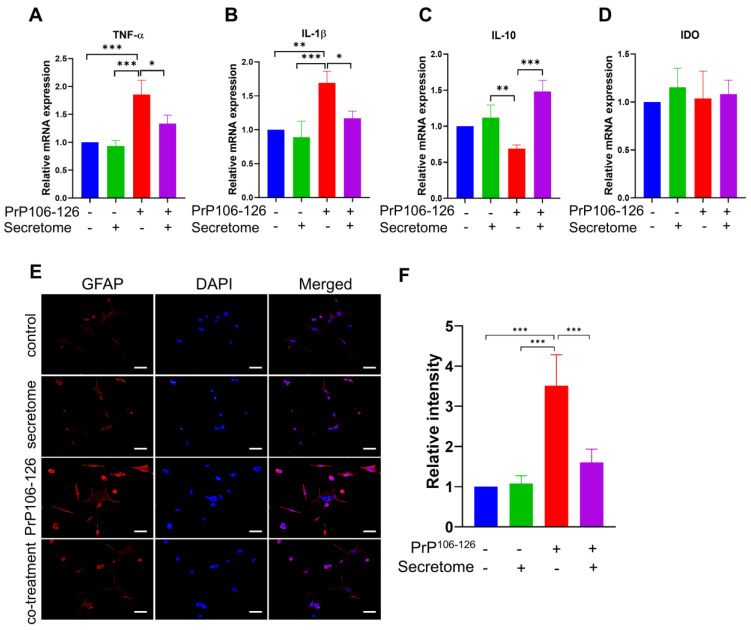
The adipose-derived mesenchymal stem cell (AdMSC) secretome mitigates neuroinflammation. (**A**–**D**) RT-PCR analysis showing that the AdMSC secretome modulates the gene expression of pro- and anti-inflammatory markers *TNF-α* (**A**), *IL-1β* (**B**), *IL-10* (**C**), and *IDO* (**D**) in SH-SY5Y cells treated with PrP^106−126^ (100 μM) and the secretome (5 µg/mL) for 24 h, compared with cells treated with PrP^106−126^ alone. Data are presented as mean ± SD (n = 3). Statistical significance was assessed using one-way ANOVA followed by post hoc Tukey’s multiple comparisons test. * *p* < 0.05, ** *p* < 0.01, *** *p* < 0.001. (**E**) Immunofluorescence images of GFAP (red) and DAPI (blue) in C8D1A astrocytic cells incubated with PrP^106−126^ (100 µM) with or without the AdMSC secretome (5 μg/mL). (**F**) Histogram representing the means ± SD (n = 3 per group). Statistical testing was performed by one-way ANOVA with post hoc Tukey’s multiple comparisons test. Scale bar = 50 μm. *** *p* < 0.001.

Reactive astrogliosis, a hallmark of inflammation, plays a significant role in the development of neuroinflammatory and neurodegenerative conditions in prion diseases [42]. To evaluate the anti-inflammatory effect of the AdMSC secretome, SH-SY5Y cells were treated with PrP^106−126^. The results showed that the secretome downregulated the gene expression of inflammatory cytokines *TNF-α* and *IL-1β* (* *p* < 0.05, Figure 2A,B). *IL-10* and *IDO* released by the MSC secretome play an immunomodulatory role [43,44]. The findings showed an upregulation of anti-inflammatory gene *IL-10* (*** *p* < 0.001) (Figure 2C) but *IDO* expression was not significantly affected (Figure 2D). Therefore, the AdMSC secretome could have a protective effect against prion-induced cytotoxicity via its anti-inflammatory activity.

PrP^106−126^ has been extensively used to model these effects, as it activates astrocytes, a central response to the neurodegenerative process [13,15,16]. To further investigate this effect, we performed an in vitro study using astrocytic C8D1A cells that were treated with PrP^106−126^ in the presence and absence of the AdMSC secretome (5 µg/mL). Following a 24 h incubation, the cells were processed for immunofluorescence staining targeting GFAP. The results and quantifications shown in Figure 2E,F indicated that AdMSC secretome treatment significantly attenuated GFAP immunoreactivity as compared to PrP^106−126^ only-treated cells (*** *p* < 0.001).

### 3.3. AdMSC Secretome Ameliorates Neuroinflammation

**Figure 3:**
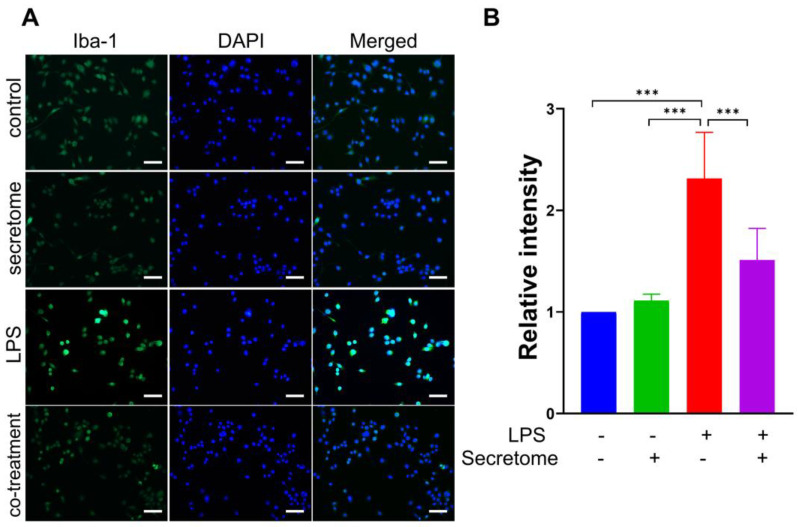
The adipose-derived mesenchymal stem cell (AdMSC) secretome reduces activated microgliosis. (**A**) Immunofluorescence staining of Iba-1 (green) in microglial BV2 cells treated with LPS (1 µg/mL) in the presence or absence of the AdMSC secretome (5 µg/mL) for 24 h. (**B**) Histogram representing the means ± SD. Statistical analysis was performed using one-way ANOVA with Tukey’s post hoc test. Scale bar = 50 μm, n = 3 per group. *** *p* < 0.001.

To evaluate the effect of the AdMSC secretome on microglial activation and neuroinflammation, we assessed its anti-inflammatory potential in the LPS-mediated microglial activation model. LPS effectively induces a robust pro-inflammatory response in microglia, mimicking the neuroinflammatory response observed in prion diseases [45]. The murine microglial cell line BV2 was treated with LPS (1 µg/mL), with or without the AdMSC secretome (5 µg/mL), for 24 h, after which immunofluorescence staining was carried out. The results showed that the AdMSC secretome reduced the immunoreactivity of Iba-1 in LPS-activated BV2 cells (Figure 3A,B; *** *p* < 0.001). Collectively, these findings implied that the AdMSC secretome may inhibit neuroinflammation in prion diseases.

### 3.4. AdMSC Secretome Inhibits Apoptosis in SH-SY5Y Cells

**Figure 4:**
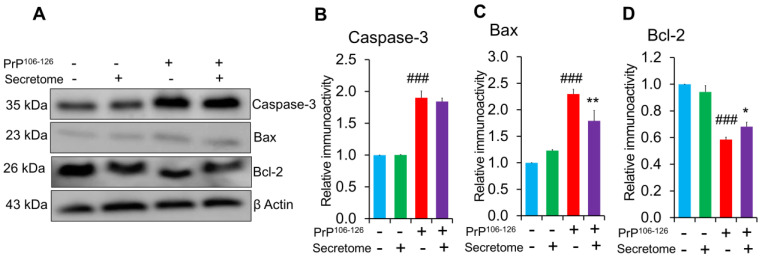
Effect of the adipose-derived mesenchymal stem cell (AdMSC) secretome on apoptosis in SH-SY5Y cells challenged with PrP^106−126^. (**A**) Protein levels of caspase-3, Bax, and Bcl-2 in SH-SY5Y cells treated with 100 μM PrP^106−126^ and 5 µg/mL of the AdMSC secretome for 24 h, as determined by western blot analysis. (**B**–**D**) Quantitative analyses of caspase-3, Bax, and Bcl-2 immunoblots, respectively. Data are presented as mean ± SD, (n = 3). β-Actin was used as a loading control. Statistical analysis was performed using one-way ANOVA with Tukey’s post hoc test. ### *p* < 0.001 vs. the control (non-exposed group), * *p* < 0.05 and ** *p* < 0.01 vs. PrP^106−126^-treated group.

To investigate the potential protective effects of the AdMSC secretome on PrP^106−126^-induced apoptosis, expression levels of pro-apoptotic markers Bax and caspase-3, as well as anti-apoptotic marker Bcl-2, were analyzed using western blotting. Altered Bax/Bcl-2 expression ratios reflect mitochondrial dysfunction, a key driver of apoptotic cell death in prion-associated neurodegeneration. Caspase-3 expression, another indicator of cell apoptosis, significantly increased in PrP^106−126^-treated SH-SY5Y cells (*** *p* < 0.001). However, treatment with the AdMSC secretome did not significantly alter caspase-3 expression levels (Figure 4A,B). The results revealed that PrP^106−126^ treatment significantly upregulated the expression of pro-apoptotic protein Bax in SH-SY5Y cells (*** *p* < 0.001), while co-treatment with the AdMSC secretome mitigated the PrP^106−126^ effect (** *p* < 0.01) (Figure 4A,C). Treatment with the AdMSC secretome also upregulated the expression of anti-apoptotic protein Bcl-2 compared to the PrP^106−126^-treated group (* *p* < 0.05) (Figure 4A,D).

### 3.5. AdMSC Secretome Enhances the Migratory Activity of SH-SY5Y Cells

**Figure 5:**
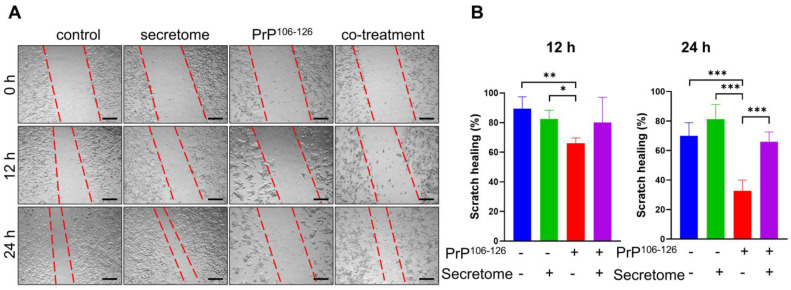
The adipose-derived mesenchymal stem cell (AdMSC) secretome enhances migratory activity. (**A**) Scratch assay of SH-SY5Y cells over 24 h. Images were taken at 0, 12, and 24 h after scratching. Scale bars = 100 µm. (**B**) Statistical analysis of wound healing was performed after 12 and 24 h using one-way ANOVA followed by post hoc Tukey’s multiple comparisons test. * *p* < 0.05, ** *p* < 0.01, *** *p* < 0.001. All data are presented as mean ± SD (n = 3).

Neurite regeneration is primarily affected by cell migration and remodeling processes [46]. The scratch assay was conducted to assess the migration properties of SH-SY5Y cells after AdMSC secretome treatment. As shown in Figure 5A, the AdMSC secretome had an effect in regulating cell migration. Quantitative analysis revealed a significant increase in the migration of SH-SY5Y cells cultured with the AdMSC secretome for 24 h compared to the PrP^106−126^-treated group (*** *p* < 0.001) (Figure 5B; n = 3). However, no significant difference was observed between groups at 12 h (Figure 5B; n = 3).

## 4. Discussion

The substances released by the stem cell secretome contain important elements such as microvesicles, exosomes, proteins, and cytokines, which may offer therapeutic potential for neurodegenerative disorders [47]. The results of the present study showed that treatment with the AdMSC secretome restored the viability of SH-SY5Y cells compromised by PrP^106−126^ exposure. The AdMSC secretome was found to reduce neuroinflammation and inhibit cellular apoptosis. Furthermore, the AdMSC secretome enhanced the migration activity of SH-SY5Y cells under PrP^106−126^ exposure. These findings suggest that the AdMSC secretome has neuroprotective effects against PrP^106−126^-induced neurotoxicity in SH-SY5Y cells.

MSCs, due to their multipotent and immunomodulatory properties, have the potential to support the recovery of different tissue functions, including in neurodegenerative diseases [48,49,50]. Adipose tissue is more readily obtainable and can be harvested frequently under local anesthesia with minimal discomfort for patients. It also contains a higher concentration of MSCs compared to bone marrow [51]. Although the success of MSCs in clinical applications has traditionally been attributed to their differentiation and migration abilities, it is now well established that MSCs mainly mediate their beneficial effects through the release of soluble paracrine bioactive molecules and EVs, particularly via their secretome [52]. Numerous studies have indicated that the MSC secretome shows promise as a treatment method for neurodegenerative diseases in both clinical and preclinical contexts (reviewed in [41,53]). Therefore, collecting the secretome from allogeneic MSCs and using it as a therapeutic strategy may be more advantageous than using MSCs themselves. In the field of chronic neurodegenerative disorders, such as prion diseases, there is a lack of in vitro or in vivo studies investigating the use of the MSC secretome as a potential therapy. In the current in vitro prion disease model, we investigated the protective role of the AdMSC secretome against PrP^106−126^.

Cytotoxicity is triggered by PrP^106−126^, which is often utilized to replicate changes associated with prion diseases in vitro [13]. Our results indicated that the application of the AdMSC secretome to SH-SY5Y cells restored the decreased cell viability caused by exposure to PrP^106−126^, as determined by the LDH assay. Similar results have been observed with the MSC secretome in other neurodegenerative disease models [53,54].

PrP^C^ is known to mediate neuroinflammation by regulating of pro- and anti-inflammatory cytokines [55,56] and has been shown to defend organs against inflammatory responses through its immunomodulation activity [57]. By contrast, increasing evidence indicates that the development of PrP^Sc^ is associated with neuroinflammation and neurotoxicity [6,7]. Immunohistochemical analyses have shown that microglia and astrocytes are closely associated with prion diseases [12,58]. Therefore, addressing neuroinflammation and cell death seems to be an effective strategy to slow the progression of prion diseases [59]. The immunomodulation effects of the AdMSC secretome in SH-SY5Y, microglial (BV2), and astrocyte (C8D1A) cells were assessed. Considering the numerous benefits of the MSC secretome [37], we hypothesized that the AdMSC secretome would ameliorate neuroinflammation. The upregulation of pro-inflammatory cytokines *like IL-1-β* and *TNF-α* occurs as prion diseases progress [59]. Under exposure to PrP^106−126^ in SH-SY5Y cells, the AdMSC secretome downregulated the expression levels of pro-inflammatory cytokine genes *IL-1β* and *TNF-α* and upregulated the expression of anti-inflammatory cytokine gene *IL-10*. Activation of microglia and astrocytes is a hallmark of prion disorders, and it is expected to increase neurotoxicity and contribute significantly to both neuronal death and disease progression [9,10,60]. Our findings indicated that the AdMSC secretome functioned as an anti-inflammatory agent and diminished the elevated levels of GFAP in astrocytic cells exposed to PrP^106−126^. Furthermore, the AdMSC secretome displayed anti-inflammatory properties and reduced the increased levels of Iba-1 and microglial activation. Proinflammatory microglia have been identified as potentially neurotoxic species in late disease periods because they generate neuroinflammation [61]. Similar results of the downregulation of pro-inflammatory cytokines along with the upregulation of anti-inflammatory cytokines following treatment with MSCs or their secretome have been reported [62,63].

On the other hand, apoptosis induced by neuroinflammation is associated with prion disorders [64]. O’Donovan et al. reported that PrP^106−126^ induced apoptosis in SH-SY5Y cells [14]. In this study, the AdMSC secretome significantly reduced apoptosis in SH-SY5Y cells by downregulating pro-apoptotic marker Bax and upregulating anti-apoptotic marker Bcl-2 (Figure 4). Although the protein levels of caspase-3 increased in PrP^106−126^-treated cells, administration of the AdMSC secretome did not decrease caspase-3 expression. This suggests that anti-apoptotic mechanisms may not involve direct caspase-3 inhibition but rather operate through alternative pathways. Consistently, conditioned medium derived from MSCs has been shown to rescue oxidatively damaged SH-SY5Y cells by decreasing apoptosis [65,66]. In addition, our previously published study showed that the AdMSC secretome decreased the level of intracellular reactive oxygen species production in SH-SY5Y cell cultures exposed to PrP^106−126^ [35]. Attracting endogenous cells with neuroregenerative properties to the sites of neuronal damage is a key objective in neuroregenerative medicine. To explore this, we further investigated whether the AdMSC secretome enhanced the migratory activity of SH-SY5Y cells under PrP^106−126^ treatment. The results showed that SH-SY5Y cells treated with the AdMSC secretome exhibited increased migratory activity compared to PrP^106−126^-treatment only, suggesting that the AdMSC secretome acts as a chemoattractant and may aid in the regeneration of neurons damaged by prion disease.

A limitation of this study is the lack of in vivo experiments to validate the observed effects of the AdMSC secretome. Therefore, additional research is required to explore the effects of the AdMSC secretome on animal models infected with prions. MSC-derived EVs are being investigated as potential therapeutic agents for neurodegenerative diseases [53]. Here, we could not elucidate the specific involvement of EVs. Thus, future studies to identify specific MSC-derived molecules that attenuate PrP^106−126^-induced neurotoxicity are required. Current evidence suggests that the secretome of AdMSCs holds significant potential as a neuroprotective treatment for prion diseases and other neuron-related conditions, including Parkinson’s disease, Alzheimer’s disease, focal cerebral ischemia, and spinal cord injuries.

## 5. Conclusions

This study demonstrates the anti-inflammatory and anti-apoptotic effects of the AdMSC secretome on SH-SY5Y neuronal cells exposed to PrP^106−126^. Treatment with the AdMSC secretome significantly enhanced SH-SY5Y cell viability while reducing LDH release, indicating diminished cytotoxicity. Furthermore, the secretome attenuated neuroinflammatory responses by markedly decreasing GFAP immunoreactivity in PrP^106−126^-treated C8D1A astrocytes (*** *p* < 0.001) and suppressing Iba-1 immunoreactivity in LPS-activated BV2 microglia (*** *p* < 0.001). Additionally, the secretome restored the impaired migratory activity of SH-SY5Y cells, suggesting a reparative effect on cellular motility. Collectively, these findings indicate that the AdMSC secretome effectively mitigates PrP^106−126^-induced neuroinflammation, apoptosis, and functional deficits. Taken together, these results highlight the therapeutic potential of the AdMSC secretome as a cytoprotective agent in regenerative medicine, offering a promising strategy to counteract neurodegenerative processes in prion diseases and related disorders.

## Figures and Tables

**Table 1:** Primer sequences used in real-time polymerase chain reaction analysis (RT-PCR).

Gene	Forward Primer	Reverse Primer
*TNF-α*	CCTCTCTCTAATCAGCCCTCTG	GAGGACCTGGGAGTAGATGAG
*IL-1β*	ATGATGGCTTATTACAGTGGCAA	GTCGGAGATTCGTAGCTGGA
*IL-10*	ACCTGCCTAACATGCTTCGAG	CTGGGTCTTGGTTCTCAGCTT
*IDO*	CAAAGGTCATGGAGATGTCC	CCACCAATAGAGAGACCAGG
*ACTB*	TGGCACCCAGCACAATGAA	CTAAGTCATAGTCCGCCTAGAAGCA

## Data Availability

The original contributions presented in this study are included in the article. Further inquiries can be directed to the corresponding author.
